# Tricuspid regurgitation and heart failure: the fate of treated vs. untreated cohort in the percutaneous era

**DOI:** 10.1093/ehjimp/qyae080

**Published:** 2024-08-05

**Authors:** Edoardo Zancanaro, Maria Rita Romeo, Annalisa Nardone, Andreina D’Agostino, Massimiliano Mariani, Sergio Berti

**Affiliations:** Cardiac Surgery Department, Heart Valve Center, San Raffaele University Hospital, Via Olgettina 69, 20140 Milan, Italy; Fondazione Toscana G. Monasterio, Ospedale del Cuore G. Pasquinucci, Massa, Italy; Fondazione Toscana G. Monasterio, Ospedale del Cuore G. Pasquinucci, Massa, Italy; Fondazione Toscana G. Monasterio, Ospedale del Cuore G. Pasquinucci, Massa, Italy; Fondazione Toscana G. Monasterio, Ospedale del Cuore G. Pasquinucci, Massa, Italy; Fondazione Toscana G. Monasterio, Ospedale del Cuore G. Pasquinucci, Massa, Italy

**Keywords:** tricuspid regurgitation, heart failure, transcatheter edge-to-edge repair, TTVR

## Abstract

**Aims:**

Tricuspid regurgitation (TR) is associated with heart failure (HF) and reduced survival. Within a short-time period, transcatheter tricuspid valve repair or replacement (TTVR/TTVr) for TR has evolved from innovation to clinical reality. The present study’s aim is to provide 1-year results between TR patients treated with TTVR and TTVr compared with untreated patients, in terms of cardiac survival, rehospitalization, right-side HF symptom development, and New York Heart Association (NYHA) improvement.

**Methods and results:**

Seventy-seven patients (pts) have been prospectively inserted into a dedicated database from January 2020 till January 2023. Twenty-six patients (33.8%) have been treated with TTVR/r [treated group (TG)], and 51 pts (66.2%) have been left untreated with medical therapy optimization [untreated group (UNTG)]. Analysing the cardiac death between the two groups, there was a significant statistical difference since TG has less incidence of exitus in the general population (*P* = 0.05). Concerning HF hospitalization, TG has a lower incidence with a *P* = 0.005. In TG, there was a significant improvement in NYHA class at follow-up (FUP) (*P* = 0.001) as well as an improvement in right-side HF symptoms (*P* = 0.001).

**Conclusion:**

This study shows that treatment in the case of TR with right-side HF has a positive impact on cardiac death and HF hospitalization at 1 year. And there is a significant improvement in clinical and echocardiographic status at FUP in the TG.

## Graphical Abstract

**Graphical Abstract:**
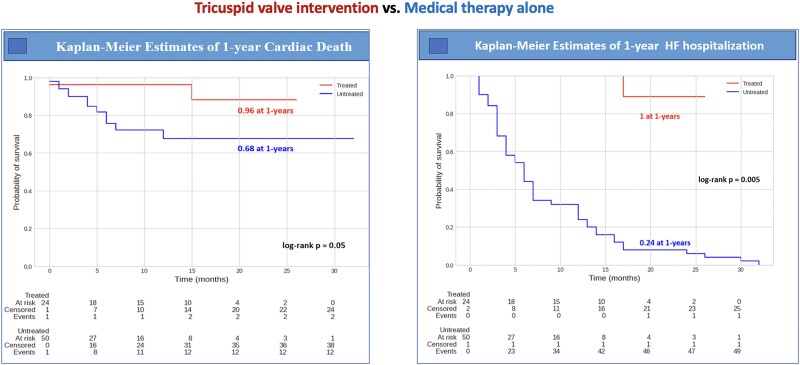

## Introduction

The tricuspid valve (TV) is often referred to as the ‘forgotten valve’ due to the initial assessment of the role of functional tricuspid regurgitation (TR) as a bystander that improved when concomitant mitral valve disease was adequately treated.^1^ Nowadays, more focus has been posed, and different treatment modalities have been implemented. What is also known is that the patients tended to present late in the course of right-side heart failure (HF), and the majority were already in the most advanced stages. The reported rate of in-hospital mortality following TR surgery is highly variable amongst different studies (10–38% of in-hospital mortality).^2,3^ Delayed referrals may account for this variability, thus highlighting the importance of optimal timing for intervention.^4^ A growing number of therapeutic alternatives such as low-risk transcatheter repair or replacement have been developed in patients not eligible for surgery.^5,6^ Devices now available can be classified according to the therapeutic target as follows: leaflets devices, annuloplasty devices, heterotopic caval valve implantation, and TTVr. They are recently included in the current European guidelines for secondary TR.^7^ However, the strength of recommendation is weak (class IIB, level of recommendation C), as their role in the management of severe TR still needs to be fully elucidated and the characteristics of the potential candidates for these techniques are not still clear. The aim of the present study is to clarify the effective results of the aforementioned devices as well as the different fate of treated and not treated patients.

## Methods

### Ethical statement

The Monasterio Hospital Institutional Ethic Committee approved this study on tricuspid insufficiency and waived individual consent for this retrospective analysis.

### Study design and patient cohort

**Figure 1:**
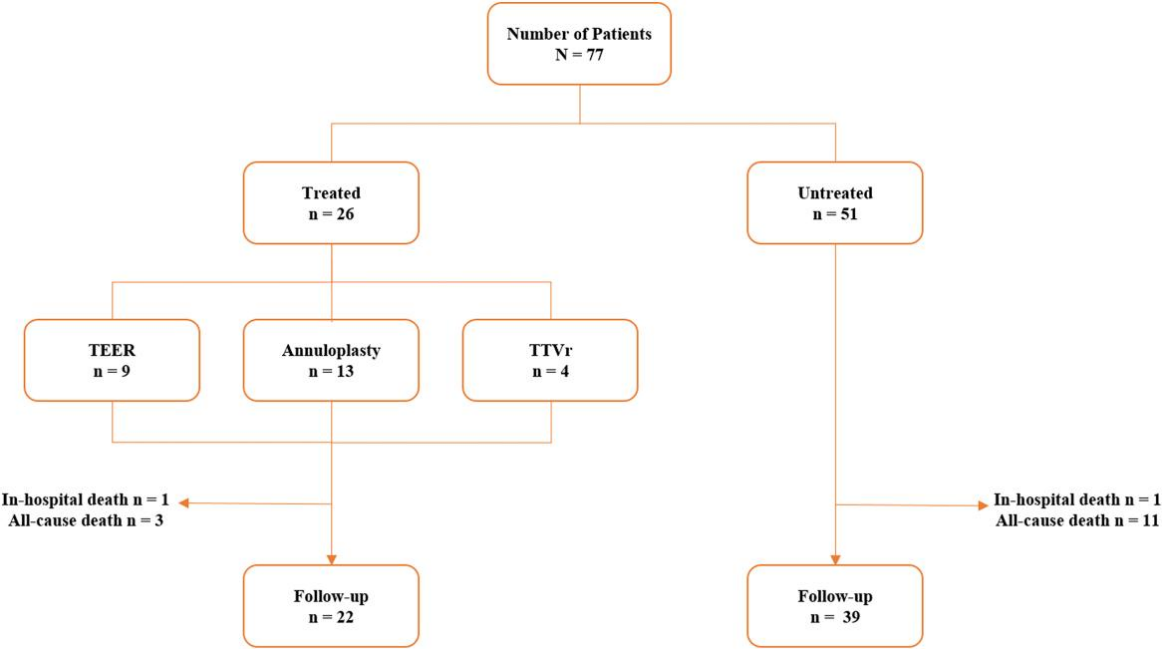
Cohort flowchart. The figure describes the total cohort distributions detailed in the text.

Seventy-seven patients (pts) have been prospectively inserted into a dedicated database from January 2020 till January 2023. Twenty-six patients (33.8%) have been treated [treated group (TG)] with transcatheter TV repair or replacement (TTVR or TTVr), and 51 pts (66.2%) have been left untreated with medical therapy (MT) optimization [untreated group (UNTG)]. Treatment was indicated according to international guidelines for TR and local heart team consensus in all patients (*Figure 1*).

Fifty-one patients (66.2%) untreated (UNTG) had optimal MT (OMT) and were judged not eligible for surgical replacement because of high and prohibited surgical risk by the local heart team. Transcatheter options were not feasible for anatomical and frailty reasons.

The collection of data was performed in line with local ethical regulations and adhered to international rules for scientific studies as well as the principles of the World Medical Association Declaration of Helsinki. Patients needed to be of legal age and able to consent. The inclusion of patients in this study was approved as required by the local Institutional Review Board and ethics committee.

The transcatheter devices for TTVR/r, included in the database, consisted of MitraClip (Abbott Vascular, Santa Clara, CA), TriClip (Abbott Vascular, Santa Clara, CA), Pascal (Edwards Lifesciences, Irvine, California), Cardioband (Edwards Lifesciences, Irvine, California), TricValve (OrbusNeich Medical Company Ltd and P&F Products Features Vertriebs), Cardiovalve (Cardiovalve Ltd), and Sapien 3 (Edwards Lifesciences, Irvine, California) (*Figure 1*; Graphical Abstract).

### Study endpoints

The aim of present study is to provide 1-year results between TR patients treated with TTVR and TTVr compared with untreated patients, in terms of cardiac survival, rehospitalization, right-side HF symptoms development, improvement of New York Heart Association (NYHA) functional class [defined as NYHA functional class improvement of at least one class at last follow-up (FUP)] and TR grade at the last FUP echocardiography. The secondary endpoint is to analyse gender disparities as well as echocardiographic and MT changes between groups.

### Definitions

TR severity and anatomic feasibility were assessed using transthoracic echocardiography (TTE) or trans-oesophageal echocardiography (TEE).

The severity of TR was assessed using a combination of semi-quantitative and quantitative assessment, as described by the American Society of Echocardiography guidelines as well as the European Association of Echocardiography guidelines.

In particular, standard 2D colour Doppler methods were used to assess TR, and TR severity was graded according to the 5-grade scheme proposed by Hahn and Zamorano.^8^

Technical success was defined differently according to the device used. In the case of transcatheter edge-to-edge repair (TEER), it was the placement of at least one clip. Concerning TTVr and other devices, it was the correct positioning without embolization or misplacement.

Procedural success was defined as the successful implantation of the device and a post-procedural TR ≤ 2.

### Statistical analysis

The study population was described with means and standard deviations (SDs) for quantitative variables and with frequencies for qualitative variables. The continuous variable distributions were tested using the Shapiro–Wilk test. Quantitative variables were compared using the Student’s *t*-test when the distribution was Gaussian and with the Mann–Whitney test, otherwise. For qualitative variables, groups were compared using the *χ*^2^ test or Fisher’s exact test.

Cumulative survival and freedom from unplanned hospitalization for HF were analysed using Kaplan–Meier models, and comparisons were performed using the log-rank test. Statistical analysis was performed with the statistical programming language R version 3.2.1 (R Foundation for Statistical Computing, Vienna, Austria) and Python Software Foundation (Python Language Reference, version 3.7).

A *P* < 0.05 was considered to indicate statistical significance, and all reported *P*-values are two-sided.

## Results

**Figure 2:**
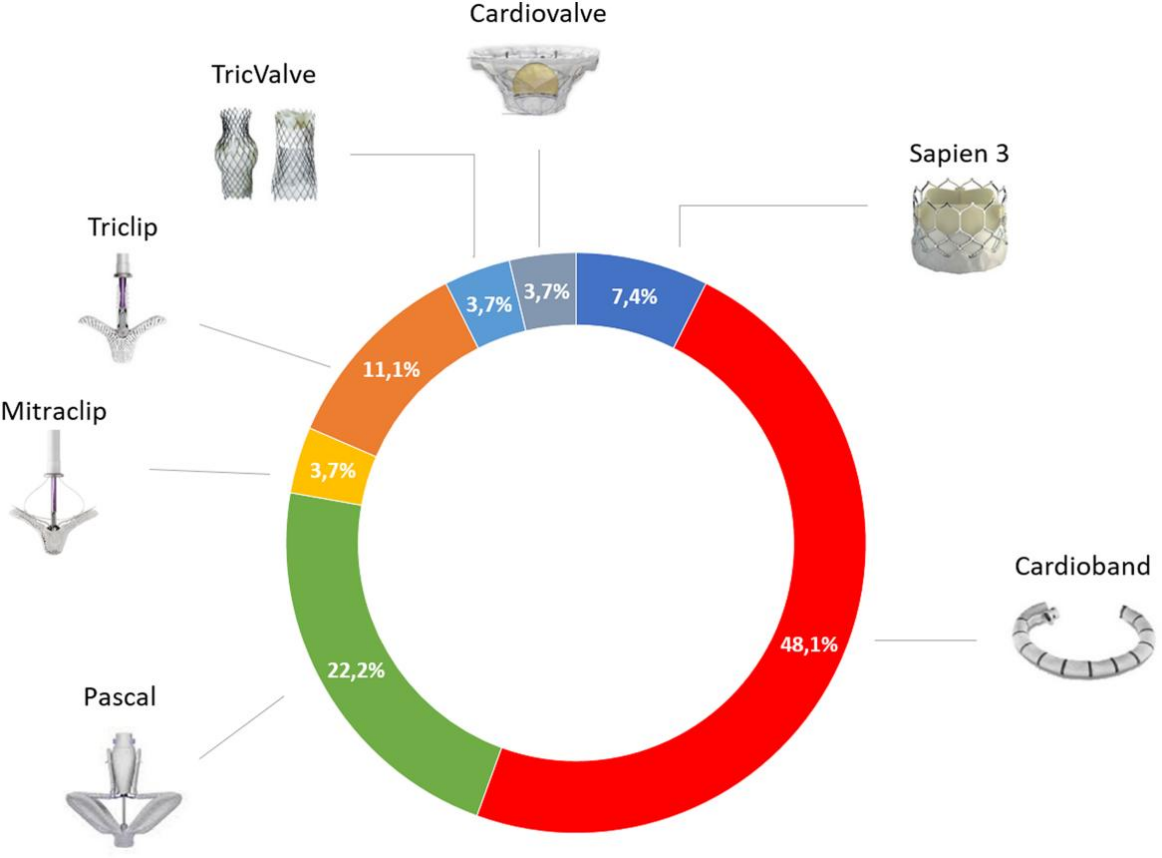
Device distribution. The figure describes the distribution of all devices used in the patient treated cohort.

Of the 77 patients referred for TR, 26 pts (33.8%) have been treated with TTVR/r (TG) and 51 pts (66.2%) have been left untreated with MT optimization (UNTG). In TG, 9 (34.6%) underwent TEER, 13 (50.0%) annuloplasty, and 4 (15.4%) TTVr (*Figure 2*).

Patients’ baseline characteristics are summarized in *Table 1*.

**Table 1:** Baseline characteristics

	Overall (*n* = 77)	Treated (*n* = 26)	Untreated (*n* = 51)	*P*-value
Baseline demographics				
Age (years)	77.9 ± 9.2	77.4 ± 8.5	78.3 ± 9.6	>0.05
Female	47 (61.0)	20 (76.9)	27 (52.9)	**<0.05**
Body mass index (kg/m^2^)	24.0 ± 5.5	24.7 ± 3.6	23.7 ± 6.2	>0.05
EuroSCORE II (%)	7.2 ± 3.6	4.4 ± 3.0	8.6 ± 3.1	**<0.001**
TRI-SCORE	5.4 ± 2.1	3.9 ± 1.7	6.2 ± 1.9	**<0.001**
NYHA functional class				**0.01**
I	0 (0.0)	0 (0.0)	0 (0.0)	
II	16 (20.8)	1 (3.9)	15 (29.4)	
III	53 (68.8)	23 (88.5)	30 (58.8)	
IV	8 (10.4)	2 (7.7)	6 (11.8)	
TR aetiology				
Functional				
Atrial	43 (55.8)	20 (76.9)	23 (45.1)	**0.008**
Ventricular	33 (42.9)	5 (19.2)	28 (54.9)	**0.003**
Degenerative	0 (0.0)	0 (0.0)	0 (0.0)	
Mixed	0 (0.0)	0 (0.0)	0 (0.0)	
PM-induced	1 (1.3)	1 (3.9)	0 (0.0)	>0.05
Comorbidities and risk factors				
Previous intervention (surgical/transcatheter)	33 (42.9)	21 (80.8)	12 (23.5)	**<0.001**
Diabetes	17 (22.1)	6 (23.1)	11 (21.6)	>0.05
Smoke	11 (14.3)	5 (19.2)	6 (11.8)	>0.05
Hypertension	41 (53.2)	21 (80.8)	20 (39.2)	**<0.001**
Dyslipidaemia	16 (20.8)	6 (23.1)	10 (19.6)	>0.05
Chronic renal failure	15 (19.5)	7 (26.9)	8 (15.7)	>0.05
Jugular turgor	24 (31.2)	10 (38.5)	14 (27.5)	>0.05
Ascites	7 (9.1)	5 (19.2)	2 (3.9)	**0.02**
Inappetence	14 (18.2)	8 (30.8)	6 (11.8)	**0.04**
Dialysis	2 (2.6)	1 (3.9)	1 (1.9)	>0.05
Carotid disease	2 (2.6)	2 (7.7)	0 (0.0)	**<0.05**
Cerebrovascular disease (stroke or TIA)	7 (9.1)	5 (19.2)	2 (3.9)	**0.03**
Previous myocardial infarction	1 (1.3)	1 (3.9)	0 (0.0)	>0.05
Right congestive HF	18 (23.4)	6 (23.1)	12 (23.5)	>0.05
COPD	14 (18.2)	8 (30.8)	6 (11.8)	**<0.05**
Coronary artery disease	7 (9.1)	2 (7.7)	5 (9.8)	>0.05
Endocarditis	0 (0.0)	0 (0.0)	0 (0.0)	
Previous PM/ICD/CRT	20 (25.9)	11 (42.3)	9 (17.7)	**<0.05**
Atrial fibrillation	40 (51.9)	21 (80.8)	19 (37.3)	**<0.001**

Values are mean ± SD or *n* (%). Bold values are statistically significant.

EuroSCORE, European System for Cardiac Operative Risk Evaluation; TRI-SCORE, Risk Score Model for Isolated Tricuspid Valve Surgery; NYHA, New York Heart Association; TIA, transient ischaemic attack; COPD, chronic obstructive pulmonary disease; PM, pacemaker; ICD, implantable cardioverter defibrillator; CRT, cardiac resynchronization therapy.

Most of the patients were in class NYHA III with 88.5% in TG and 58.8% in UNTG. The European System for Cardiac Operative Risk Evaluation (EuroScore II) and Risk Score Model for Isolated Tricuspid Valve Surgery (TRI-SCORE) were higher in UNTG (8.6 ± 3.1% and 6.2 ± 1.9, respectively) than in TG (4.4 ± 3.0% and 3.9 ± 1.7, respectively; *P* < 0.001).

TG showed a worse clinical setting having higher hypertension (80.8%), ascites (19.2%), inappetence (30.8%), and chronic obstructive pulmonary disease (30.8%). Female gender were 52.9% in UNTG while 76.9% in TG (*P* < 0.05; *Table 1*).

According to the TR stage at baseline, 42.3% of the TG patients were in stage 4, while in UNTG, 49.0% of the patients were in stage 5 (Supplementary data online, *Figure S1*).

### Baseline echocardiographic values

TR aetiology was predominantly functional as expected with one case of pacemaker-induced TR. Atrial functional TR (atrial FTR) was represented in 55.8% of the total cohort with a higher prevalence in the case of TG (76.9%) while ventricular form was more prevalent in UNTG (54.9%). And atrial fibrillation was higher in TG (80.8%) (*Table 1*).

Pre-procedural TR severity was predominantly torrential and severe in 37.7% and 28.6% of the total cohort, respectively; in TG, it was mostly torrential (65.4%) and predominantly severe in UNTG (41.2%). Nevertheless, TR effective regurgitant orifice area was higher in TG than in UNTG (0.7 ± 0.3cm² vs. 0.5 ± 0.1cm²; *P* < 0.001; *Table 2*).

**Table 2:** Baseline echocardiographic parameters

	Treated (*n* = 26)	Untreated (*n* = 51)	*P*-value
TR severity			**<0.001**
0 = none/trace	0(0.0)	0(0.0)	
1 = mild	0(0.0)	0(0.0)	
2 = moderate	0(0.0)	5 (9.8)	
3 = severe	1 (3.8)	21 (41.2)	
4 = massive	8 (30.8)	13 (25.5)	
5 = torrential	17 (65.4)	12 (23.5)	
Tricuspid EROA (cm^2^)	0.7 ± 0.3	0.5 ± 0.1	**<0.001**
Tricuspid vena contracta (mm)	11.3 ± 3.3	10.1 ± 1.5	>0.05
LVEF (%)	55.1 ± 10.4	51.8 ± 9.9	>0.05
RVEF (%)	36.3 ± 4.9	36.4 ± 4.9	>0.05
RVEDD (mm)	43.7 ± 3.7	43.9 ± 3.8	>0.05
LVEDD (mm)	45.3 ± 5.3	52.8 ± 6.6	**<0.001**
RV FAC (%)	32.7 ± 6.1	32.9 ± 8.4	>0.05
TAPSE (mm)	18 ± 3.7	18.6 ± 4.2	>0.05
S-TDI (cm/s)	11.5 ± 4.6	10.2 ± 2.2	>0.05
Concomitant MR ( ≥ 3^+^)	4 (15.4)	3 (5.9)	**<0.05**
sPAP (mmHg)	42.7 ± 10.6	41.9 ± 9.3	>0.05
IVC diameter (cm)	2.4 ± 0.4	2.5 ± 0.3	>0.05

Values are mean ± SD or *n* (%). Bold values are statistically significant.

EROA, effective regurgitant orifice area; FAC, fractional area change; IVC, inferior vena cava; LVEDD, left ventricular end-diastolic diameter; LVEF, left ventricular ejection fraction; MR, mitral regurgitation; RV, right ventricular; RVEDD, right ventricular end-diastolic diameter; RVEF, right ventricular ejection fraction; sPAP, systolic pulmonary artery pressure; S-TDI, systolic tissue Doppler imaging; TAPSE, tricuspid annular plane systolic excursion; TR, tricuspid regurgitation.

Right ventricular ejection fraction was similar amongst groups (36.3 ± 4.9% vs. 36.4 ± 4.9%; *P* > 0.05) as right ventricular end-diastolic diameter (43.7 ± 3.7% vs. 43.9 ± 3.8%; *P* > 0.05), right ventricular fractional area change (32.7 ± 6.1% vs. 32.9 ± 8.4%; *P* > 0.05), and left ventricular ejection fraction (55.1 ± 10.4% vs. 51.8 ± 9.9%; *P* > 0.05; *Table 2*).

Concomitant MR ≥ 3 was seen in 7 pts with higher prevalence in TG than UNTG (15.4% vs. 5.9%; *P* < 0.05).

### Procedural results and adverse events

The technical success rate was defined differently according to the device used. In the case of TEER, it was the placement of at least one clip (100%). Concerning TTVr and other devices (Cardioband/TricValve), it was the correct positioning without embolization or misplacement (100%). Procedural success was defined as the successful implantation of the device and a post-procedural TR ≤ 2. It was 73.1%.

The procedural data are summarized in *Table 3*.

**Table 3:** Procedural characteristics (TG, *n* = 26)

Technical success^a^	26 (100)
Procedural success^b^	24 (73.1%)
Post-procedural TR severity	
0 = none/trace	3 (11.5)
1 = mild	6 (23.1)
2 = moderate	7 (26.9)
3 = severe	6 (23.1)
4 = massive	3 (11.5)
5 = torrential	1 (3.8)
Reduction of ≥1 TR grade	24 (92.3)
Numbers of clips	
1	4 (15.4)
2	5 (19.2)
Clips location (*n* = 9)	
Anteroseptal	7 (77.7)
Anteroseptal + posteroseptal	2 (22.2)
Post-procedural length of hospital stay (days)	7.2 ± 7.2

Values are *n* (%) or mean ± SD.

TR, tricuspid regurgitation.

^a^Technical success rate was defined differently according to the device used. In case of TEER, it was the placement of at least one clip. Concerning TTVr and other devices, it was the correct positioning without embolization or misplacement.

^b^Procedural success was defined as the successful implantation of the device and a post-procedural TR ≤ 2.

On average, two clips were placed, predominantly in the anteroseptal position (in 77.7% of patients solely in the anteroseptal position and in 22.2% of patients in the anteroseptal and posteroseptal positions). Clip placement/other devices decreased the proportion of patients with TR ≥ 3 from 100% pre-procedurally to 38.5% before discharge (*P* < 0.001).

Concomitant mitral valve edge-to-edge procedures, preceding the tricuspid intervention, were never performed.

There were no procedural deaths. Two patients (2.5%) died during the hospitalization. No major complications during the hospitalization and in the postoperative period were seen. One case of acute kidney injury and one of the ventricular arrhythmias were noted without sequelae (Supplementary data online, *Table S1*).

### One-year outcomes

During a median FUP period of 1 year, 14 patients (18.2%) died from the total cohort but 11/51 (21.6%) in UNTG and 3/26 (11.5%) in TG (Supplementary data online, *Table S2*).

**Figure 3:**
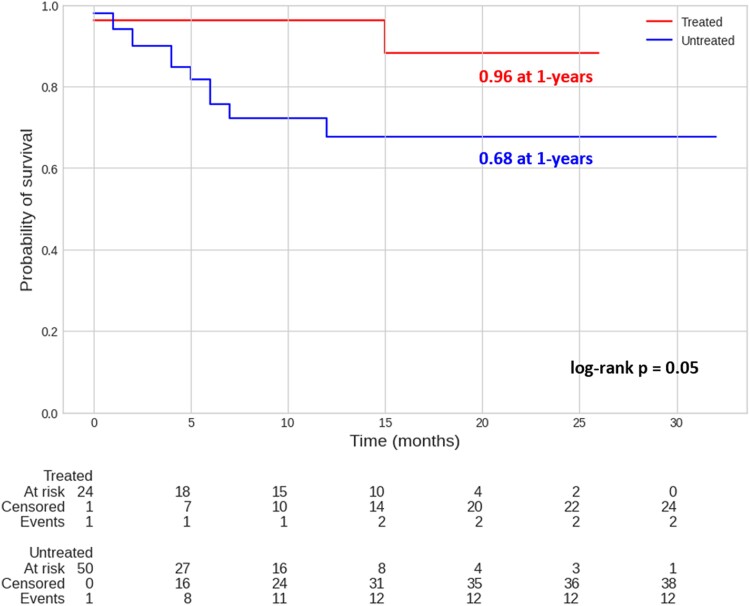
Kaplan–Meier estimates of 1-year cardiac death. Kaplan–Meier estimates for cardiac death at 1 year, stratified for TG (3.8%; 95% confidence interval: 0.5–24.3%) and UNTG (32.3%; 95% confidence interval: 19.2–51.2%).

Kaplan–Meier estimates 1-year cardiac death, which was 3.8% (95% confidence interval: 0.5–24.3%) in TG and 32.3% (95% confidence interval: 19.2–51.2%) in UNTG (*P* = 0.05; *Figure 3*).

**Figure 4:**
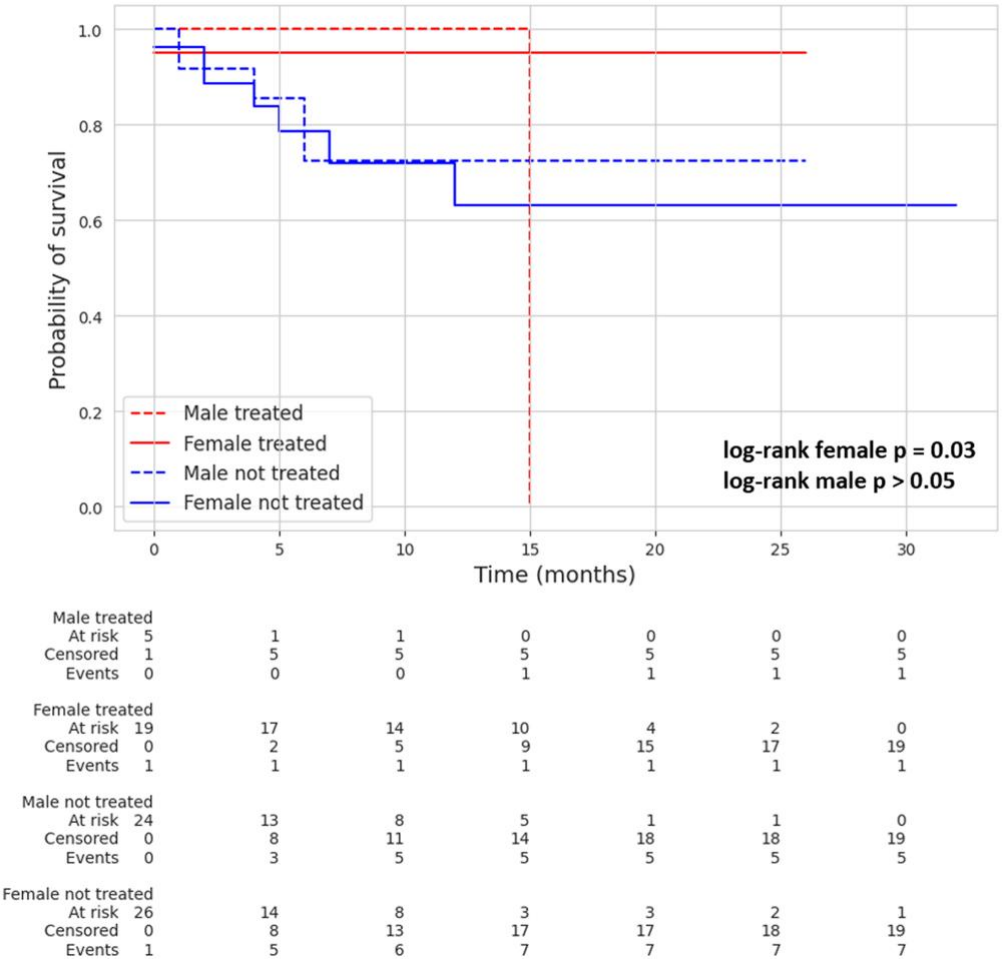
Kaplan–Meier estimates of 1-year cardiac death according to gender. Kaplan–Meier estimates for cardiac death at 1 year, stratified for female in TG and UNTG (5%; 95% confidence interval: 0.7–30.5% and 36.9%; 95% confidence interval: 18.3–64.9%), respectively) and male in TG and UNTG (0 and 27.6%; 95% confidence interval: 12.2–55.2%, respectively).

In the female gender, the patients have cardiac death incidence of 5.0% (95% confidence interval: 0.7–30.5%) in TG while 36.9% (95% confidence interval: 18.3–64.9%) in UNTG (*P* = 0.03; *Figure 4*).

**Figure 5:**
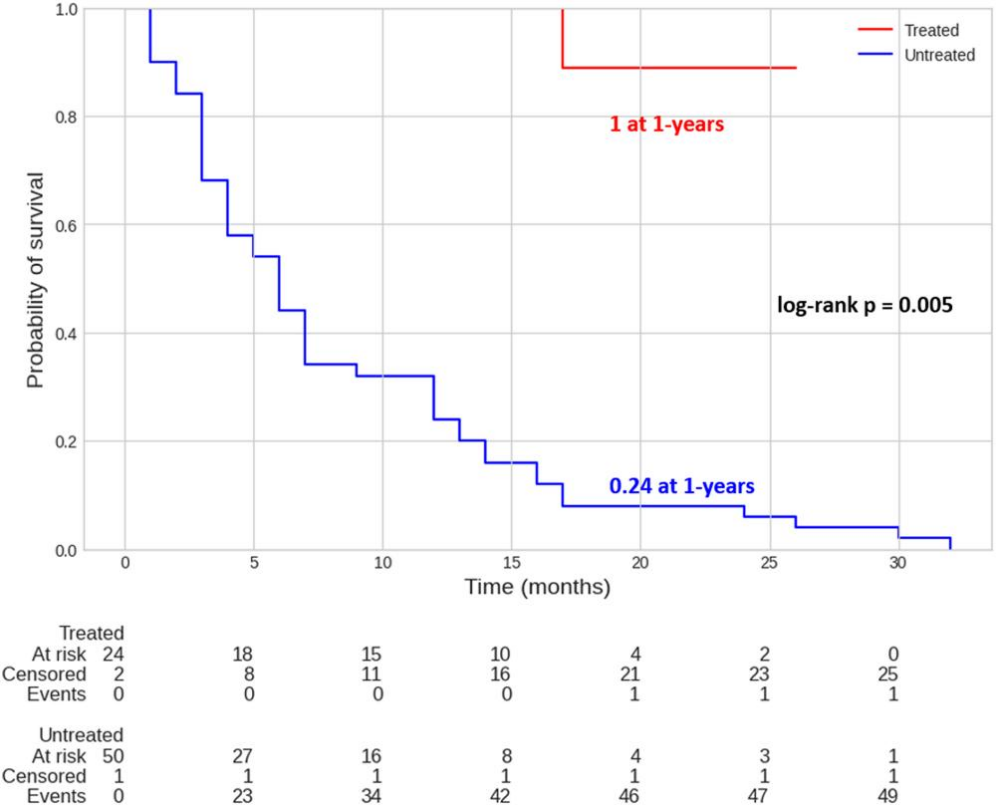
Kaplan–Meier estimates of 1-year HF hospitalization. Kaplan–Meier estimates for HF hospitalization at 1 year, stratified for TG (0%) and UNTG (76%; 95% confidence interval: 63.6–86.7%).

Kaplan–Meier estimates 1-year HF hospitalization, which was 0% in TG vs. 76.0% (95% confidence interval: 63.6–86.7%) in UNTG (*P* = 0.005; *Figure 5*).

Kaplan–Meier estimates HF hospitalization at 1 year, stratified for females in TG and UNTG, was 0% vs. 80.8% (95% confidence interval: 64.0–92.9%), respectively (Supplementary data online, *Figure S2*).

### Clinical and echocardiographic FUP

Data on clinical outcomes were available in 100% of eligible patients. In TG, there was a significant improvement in NYHA class at FUP compared with UNTG (*P* = 0.001).

**Figure 6:**
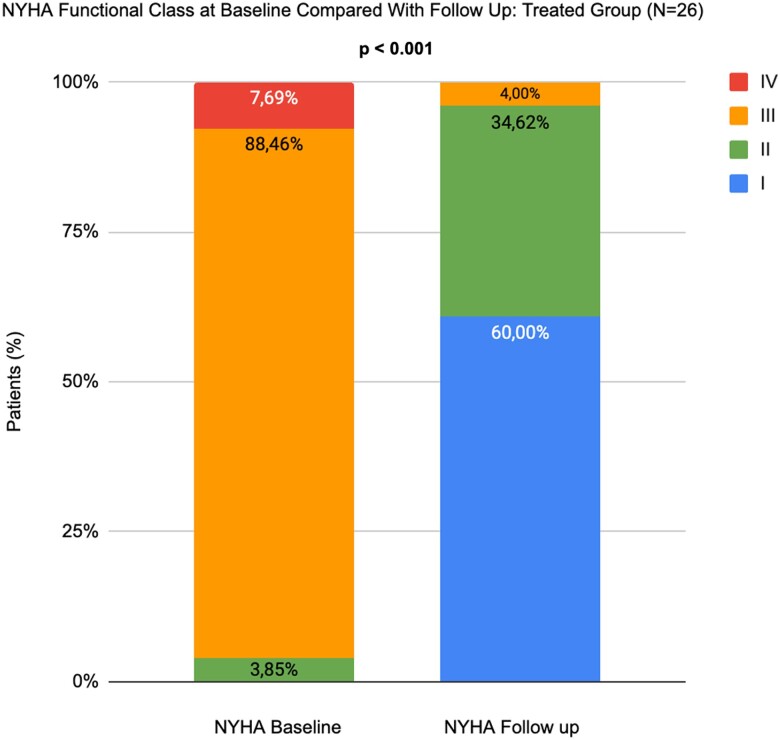
NYHA functional class at baseline compared with FUP: TG (*n* = 26). NYHA functional class at baseline and FUP. At 1 year, 15 pts (60.0%) had NYHA class I in TG. NYHA, New York Heart Association; TG, treated group.

A significant reduction of symptoms was observed in TG (3.9 and 94.6% of NYHA functional class I or II at baseline and last FUP, respectively). At 1 year, 15 pts (60%) had NYHA class I in TG while 0 pts in UNTG (*Figure 6*; Supplementary data online, *Figure S3*).

An improvement of right-side HF symptoms was also seen since ascites, inappetence, and jugular turgor were seen in 0, 4.5, and 0% respectively, in TG with *P* = 0.001, while it was seen in 28.2, 25.6, and 25.6%, respectively, in UNTG (*P* = 0.001).

In UNTG, there was a significant increase in the dosage of HF drugs (diuretics, beta-blockers, ARNI, etc.) compared with TG (*P* = 0.001) till the optimal guideline MT (GDMT) dosage.

**Figure 7:**
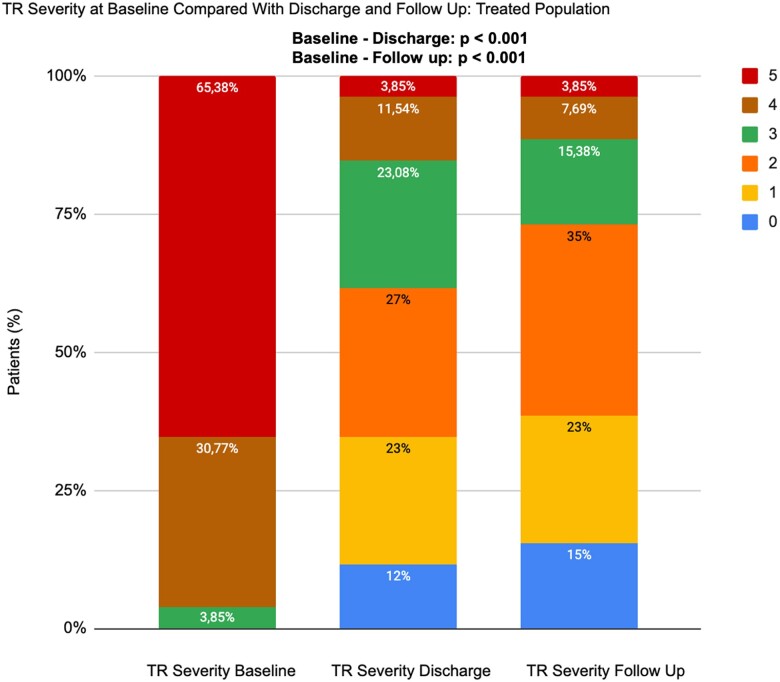
TR severity at baseline compared with discharge and FUP: TG (*n* = 26). TR severity at baseline, discharge, and last FUP in TG. The proportion of patients with TR ≥ 3 was 100% pre-procedure and 38.46% before discharge. TR, tricuspid regurgitation.

Echocardiographic FUP was available in 96% of the eligible patients. Compared with baseline echocardiography, the prevalence of TR was significantly reduced in 92.3% of treated patients at FUP (*Figure 7*; Supplementary data online, *Figure S4*).

There was an increase in right ventricular function and dimension in the case of UNTG from baseline to FUP (*P* = 0.001).

## Discussion

In this analysis from a single-centre prospective study, we investigated 1-year outcomes in patients with severe symptomatic TR and HF, either treated or left in GDMT. Patients included in this study were deemed at high or prohibitive surgical risk and were treated according to the heart team’s multidisciplinary evaluation.

The study results can be summarized as follows: (i) 1-year survival was significantly higher in the case of patients treated, with whatever device, compared with whom was left in MT; (ii) HF hospitalization rate, at 1 year, was definitely higher in the case of non-treated patients as well as the worsening of right-side HF symptoms; (iii) TG benefits from better clinical and echocardiographic outcomes in terms of NYHA changes and TR reduction at 1-year FUP; and (iv) in the female population, it was seen a significantly better survival in TG while no difference in case of HF hospitalization rate if compared with the general population.

To our knowledge, this cohort represents the first that is able to compare different TR treatment techniques with MT alone in the case of HF patients due to TR pathology, defining the fate of HF patients, in the percutaneous era.

Several considerations have to be made:

First, TR treatment with different devices has shown to be a safe procedure and free of major complications, as also demonstrated by Gupta *et al*.^9^

In our cohort, correct device positioning was seen in the total cohort of treated patients with no major complications. The in-hospital mortality, in the total cohort, is in line with general experience.^10^ Several devices have been used, and limited complications have been noted. The latest update has been by Goldberg *et al*.^11^ that analysed the impact and differences of TTVR in the case of TR concluding that TTVR devices have an advantage in many ways over both surgical TTVr and TTVR. They are less dependent on leaflet morphology or aetiology of TR than repair devices and without surgical morbidity and mortality risks associated with open heart surgery. Concerning our cohort, we did not differentiate between repair and replacement techniques, but we hereby confirmed the safety profile of these.

Secondly, concerning the impact of TR treatment, it has been mentioned that TEER represents a valid option in terms of survival. Sorajja *et al*. has shown a benefit in terms of survival in the case of treated patients (win ratio, 1.48; 95% confidence interval, 1.06–2.13; *P* = 0.02). Our cohort has proven that TG benefits from TR reduction since cardiac death was significantly lower than UNTG (*P* = 0.05). The difference with Sorajja *et al*. is based on the different devices used.^12^

What has been shown in the Triluminate trial is that the rate of hospitalization for HF did not appear to differ between the groups while it was significantly increased in our cohort for whom was left into solely MT (*P* = 0.005). This aspect can be due to the fact that multiple devices have been used and the UNTG cohort has shown a worse baseline clinical setting.^12^

On the other hand, HF patients have generally shown worse clinical outcomes in terms of survival and hospitalization rate. Our data corroborate these data since patients left in MT demonstrated worse outcomes having a 32.3% of cardiac death at 1 year with a 76.0% rate of hospitalization.^13^ The quality of life aspect has to be also considered since patients that underwent a TR reduction also experience a better class NYHA at FUP as well as a reduction of right-side HF symptoms (ascites, inappetence, peripheral oedema, and jugular turgor). Moreover, MT dosage was implemented in the case of UNTG while it was decreased in the case of TG.

Finally, despite difficulties in accurate quantitative echocardiographic TR grading, TR severity was reduced by at least 1 grade in 92.3% of the treated patients. This has been previously seen in different studies. Lurz *et al*.^14^ has shown that the results at 1 year after TR treatment are maintained durable from the discharge. The aforementioned focus on TR reduction was also seen by Alperi *et al*.^15^ saying that the future perspective is to treat severe TR before as possible trying to avoid excessive RV remodelling and dysfunction. HF progression is corroborated with worse outcomes, and patients affected by TR match the treatment too late in their medical progression. For sure, the present data, even if it is hypothesis-generating, reinforce the already present consolidated results of the superiority of TR device–mediated treatment compared with MT alone.

### Limitations

Because the data were collected in a prospective, observational, but non-randomized, and non-controlled fashion, it is not possible to discern the effect of the intervention from MT alone in patients with TR. The second important limitation is the small number of the present cohort compared with bigger clinical trials. Of notice, no propensity score matching has been used because of small numbers, but the two cohorts did not show too different pre-operative settings.

## Conclusions

TR is associated with HF and reduced survival. Within a short-time period, TTVR or TTVr for TR has evolved from innovation to clinical reality. Still, most patients arrive in an advanced state of HF, and most cohorts remained untreated for various reasons. TR treatment overcome in terms of survival and clinical status of patients left in MT with a HF status.

## Supplementary Material

qyae080_Supplementary_Data

## Data Availability

Data are available upon requested and have been supported by the team.

## Lead author biography

Edoardo Zancanaro

Address: Rasori 1, 20145, Milan, Italy

Email address: e.zancanaro96@gmail.com; Phone number: (+39) 3663537754 Gender: male; date of birth: 18/01/1996; Nationality: Italian

[From 01/01/2024 to current] Cardiac surgery and interventional cardiology fellow at University Medical Center of Johannes Gutenberg-University Mainz

Prof. H. Treede, Dr DS. Dohle, Prof. P.Lurz, Prof. RS von Bardeleben

[29/09/2022–current] Cardiac Surgery Residency—cardiac surgery resident at San Raffaele Hospital, Milan, Italy

Prof. M. De Bonis and Prof. O.Alfieri

[01/11/2021 – 8/09/2022] Interventional Cardiology Department Residency/Fellowship

Dott. Sergio Berti
