# Supplementary figures and images for: Tricuspid regurgitation and heart failure: the fate of treated vs. untreated cohort in the percutaneous era
Source: Eur Heart J Imaging Methods Pract. 2024 Aug 5;2(2):qyae080. doi: 10.1093/ehjimp/qyae080 (PMC11367954; doi:10.1093/ehjimp/qyae080)

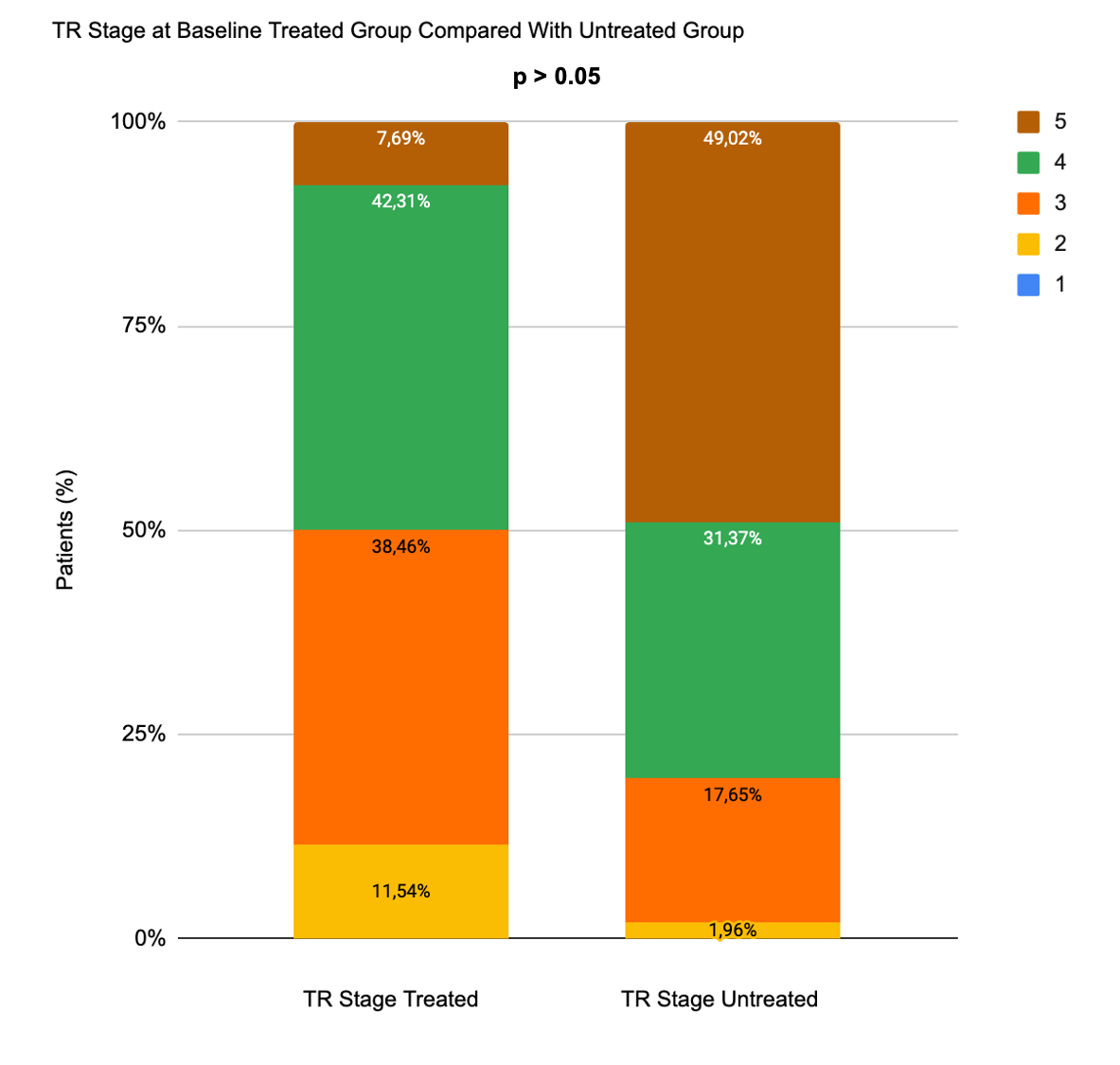

Supplement: qyae080_Supplementary_Data [file qyae080_supplementary_data.zip › Supplemental figure S1.tiff]

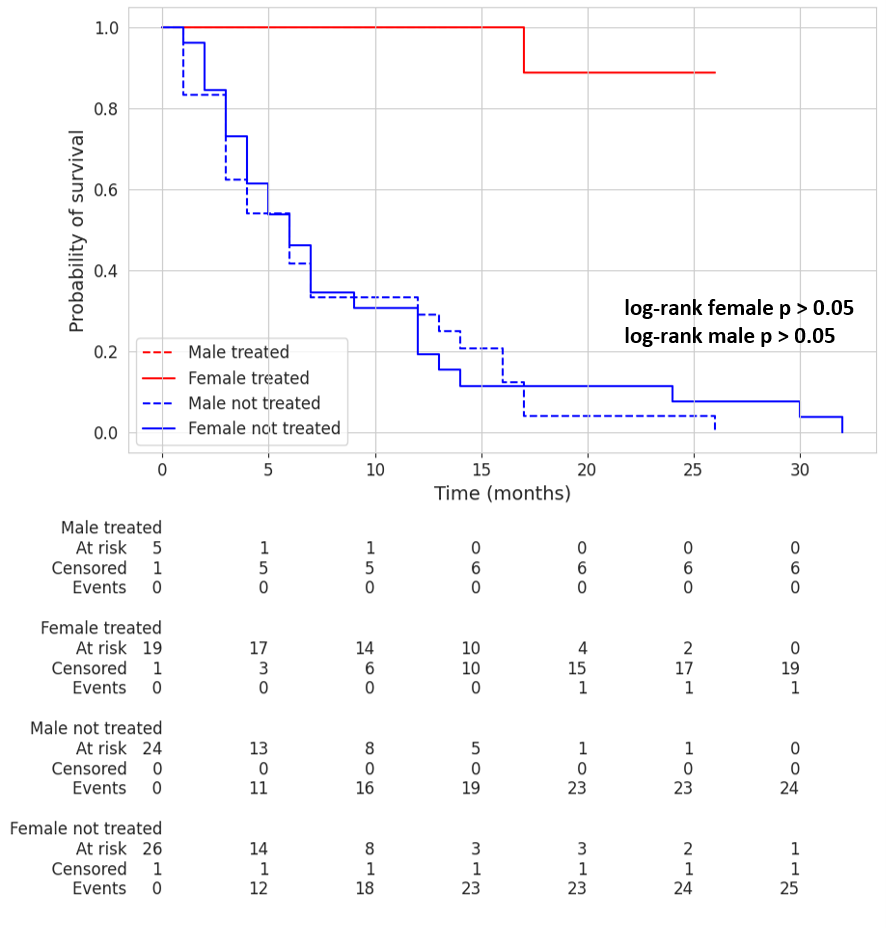

Supplement: qyae080_Supplementary_Data [file qyae080_supplementary_data.zip › Supplemental figure S2.tiff]

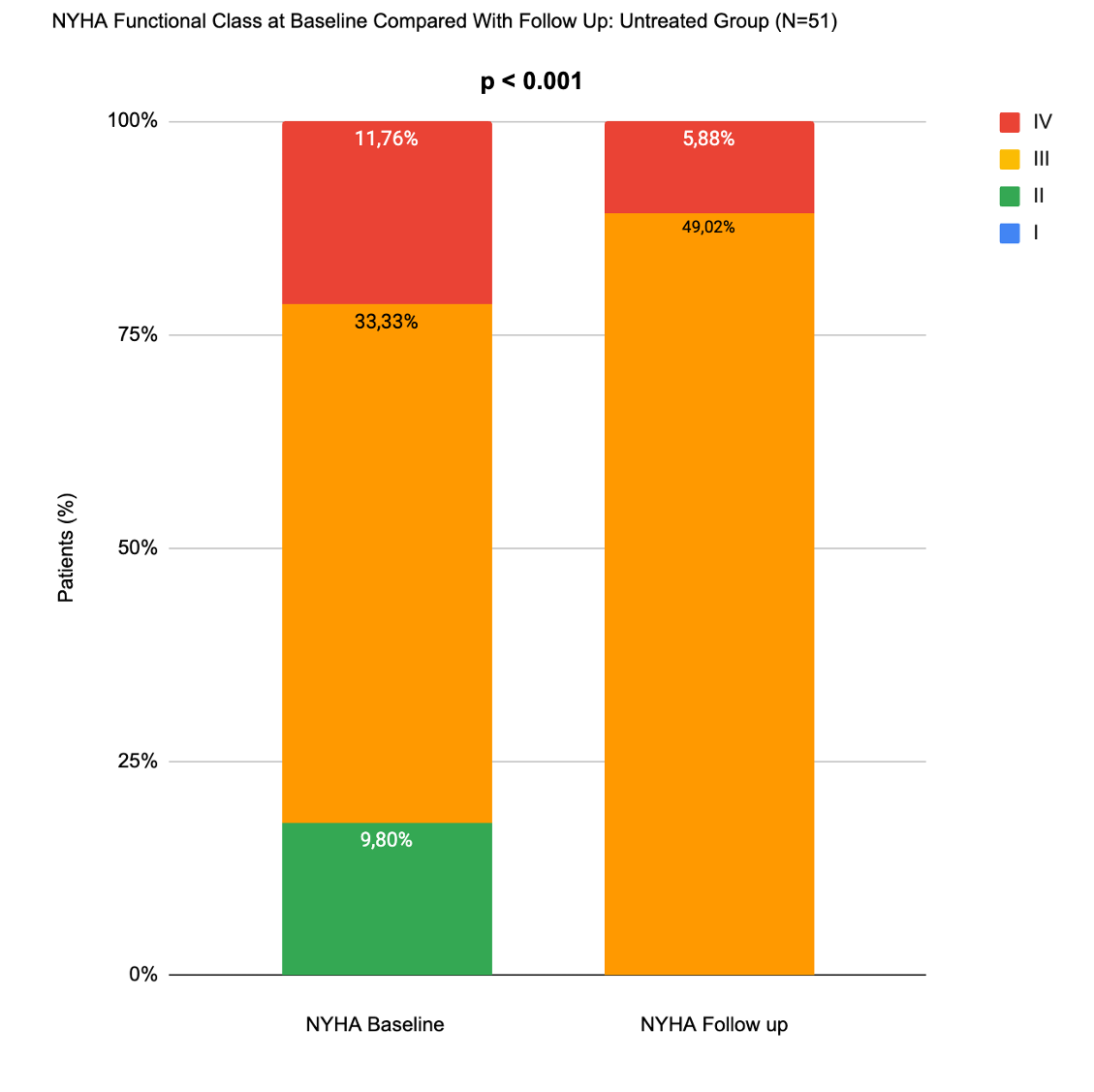

Supplement: qyae080_Supplementary_Data [file qyae080_supplementary_data.zip › Supplemental figure S3.tiff]

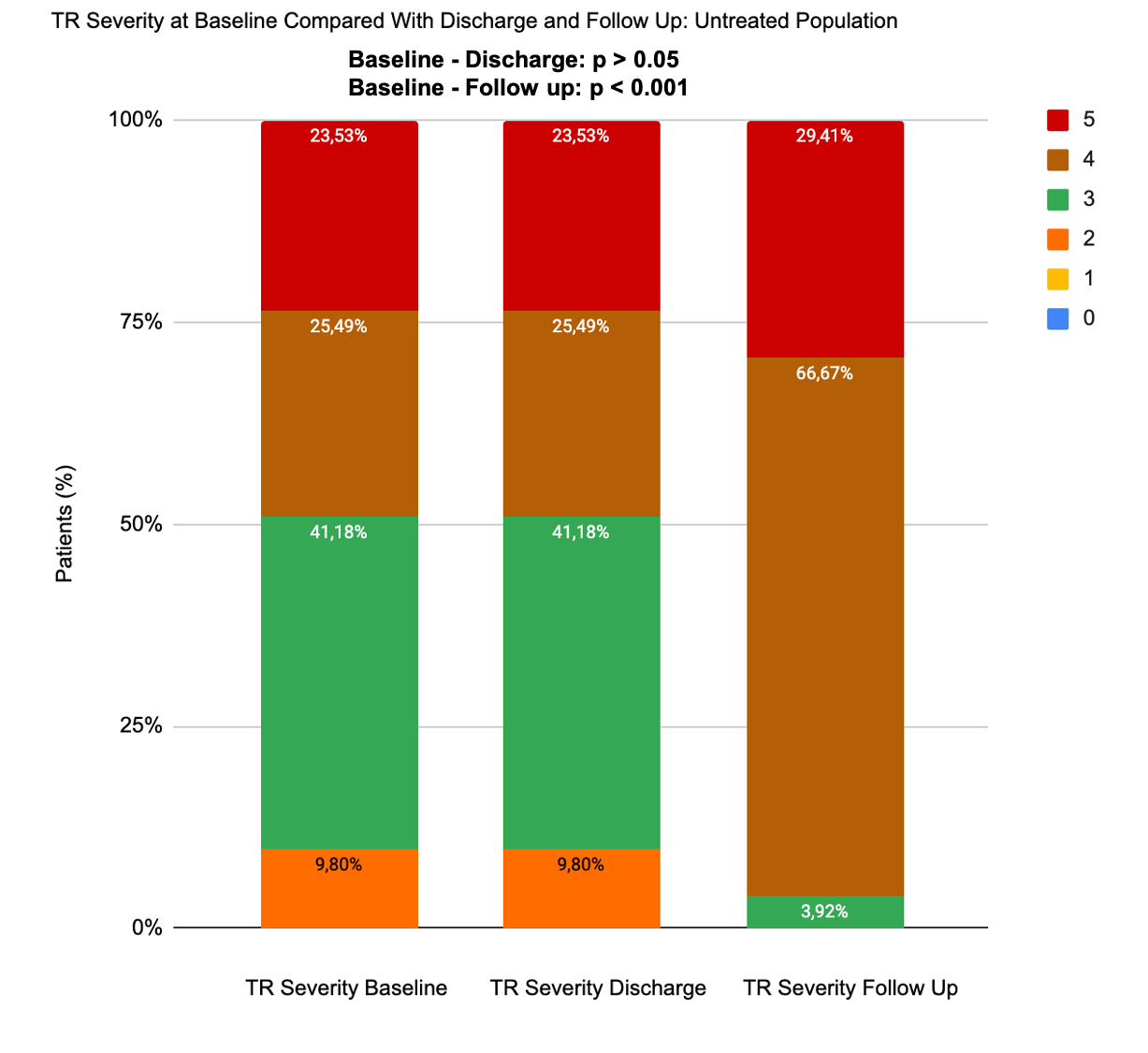

Supplement: qyae080_Supplementary_Data [file qyae080_supplementary_data.zip › Supplemental figure S4.tiff]

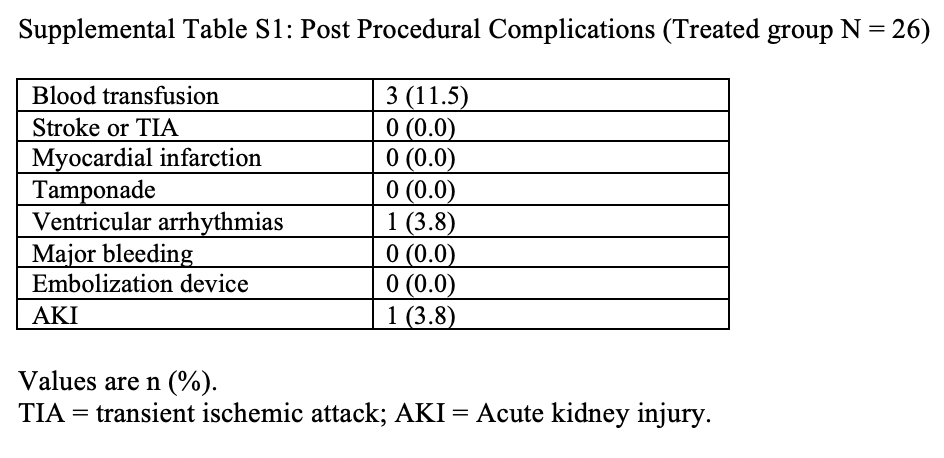

Supplement: qyae080_Supplementary_Data [file qyae080_supplementary_data.zip › Supplemental table S1.png]

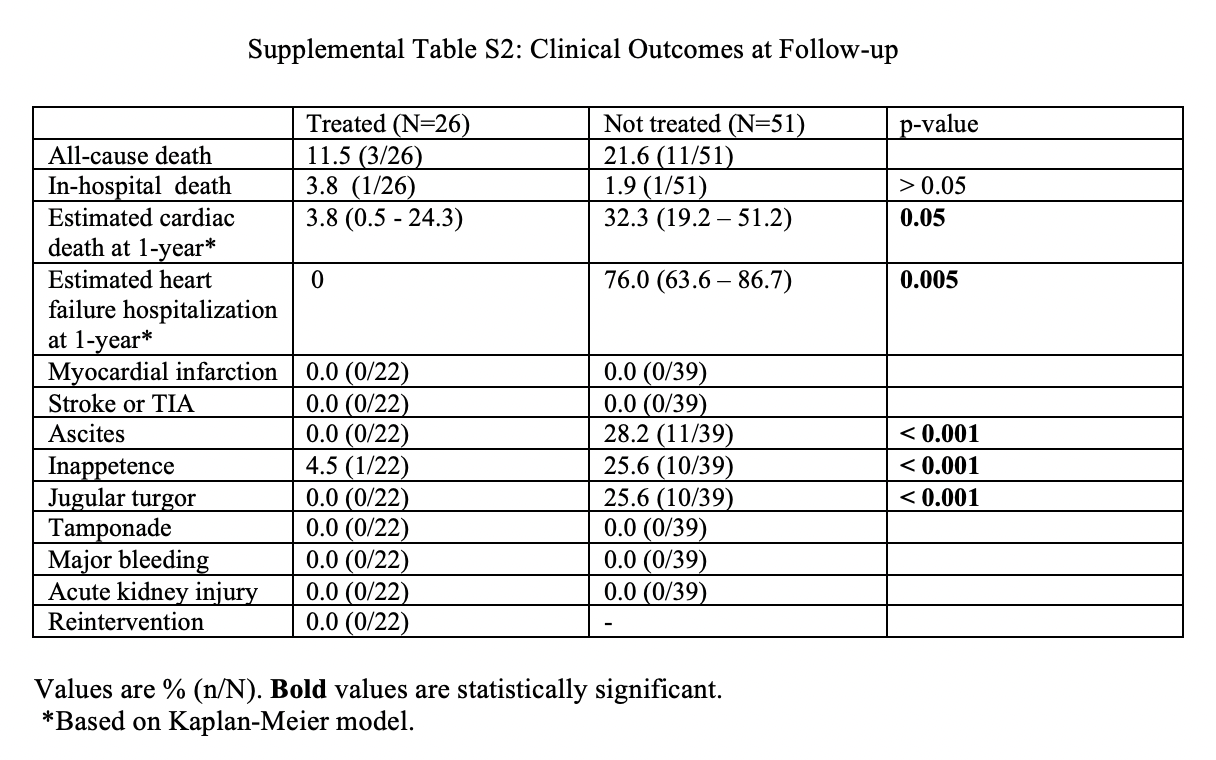

Supplement: qyae080_Supplementary_Data [file qyae080_supplementary_data.zip › Supplemental table S2.png]
